# Interlimb Coordination: A New Order Parameter and a Marker of Fatigue During Quasi-Isometric Exercise?

**DOI:** 10.3389/fphys.2020.612709

**Published:** 2021-01-12

**Authors:** Pablo Vázquez, Monika Petelczyc, Robert Hristovski, Natàlia Balagué

**Affiliations:** ^1^Complex Systems in Sport Research Group, Institut Nacional d’Educació Física de Catalunya (INEFC), Universitat de Barcelona, Barcelona, Spain; ^2^Cardiovascular Physics Group, Faculty of Physics, Warsaw University of Technology, Warsaw, Poland; ^3^Complex Systems in Sport Research Group, Faculty of Physical Education, Sport and Health, Ss. Cyril and Methodius University in Skopje, Skopje, North Macedonia

**Keywords:** order parameter, task disengagement, percolation, coordination, bivariate analysis

## Abstract

Although exercise-induced fatigue has been mostly studied from a reductionist and component-dominant approach, some authors have started to test the general predictions of theories of self-organized change during exercises performed until exhaustion. However, little is known about the effects of fatigue on interlimb coordination in quasi-isometric actions. The aim of this study was to investigate the effect of exercise-induced fatigue on upper interlimb coordination during a quasi-isometric exercise performed until exhaustion. In order to do this, we hypothesized an order parameter that governs the interlimb coordination as an interlimb correlation measure. In line with general predictions of theory of phase transitions, we expected that the locally averaged values of the order parameter will increase as the fatigue driven system approaches the point of spontaneous task disengagement. Seven participants performed a quasi-isometric task holding an Olympic bar maintaining an initial elbow flexion of 90 degrees until fatigue induced spontaneous task disengagement. The variability of the elbow angle was recorded through electrogoniometry and the obtained time series were divided into three segments for further analysis. Running correlation function (RCF) and adopted bivariate phase rectified signal averaging (BPRSA) were applied to the corresponding initial (30%) and last (30%) segments of the time series. The results of both analyses showed that the interlimb correlation increased between the initial and the final segments of the performed task. Hence, the hypothesis of the research was supported by evidence. The enhancement of the correlation in the last part means a less flexible coordination among limbs. Our results also show that the high magnitude correlation (%RCF > 0.8) and the %Range (END-BEG) may prove to be useful markers to detect the effects of effort accumulation on interlimb coordination. These results may provide information about the loss of adaptability during exercises performed until exhaustion. Finally, we briefly discuss the hypothesis of the inhibitory percolation process being the general explanation of the spontaneous task disengagement phenomenon.

## Introduction

Despite the overwhelming amount of research published over the last decades on exercise-induced fatigue^1^, little is known about its impact on performance (Enoka and Duchateau, 2016). A reductionist and component dominant approach, searching for central and peripheral mechanisms as causes of muscle force reduction during effort, could not reach clear conclusions about the specific mechanisms responsible for the phenomenon (Gandevia, 2001; Enoka and Duchateau, 2016) and the real causes of fatigue-related task failure (Hristovski and Balagué, 2010; Balagué et al., 2014).

One main characteristic of component-dominant based research on fatigue is the assumption that the variation of a single component or process can explain the whole variability of the measured task or performance output. Accordingly, the available research has been mainly oriented toward the study of continuous quantitative changes that arise at different levels (from cells to organs) during the developing fatigue. However, these continuous changes cannot, by themselves, explain the discontinuous qualitative nature of changes occurring during the process, like the spontaneous task failure or task disengagement (Hristovski and Balagué, 2010; Balagué et al., 2014).

**FIGURE 1:**
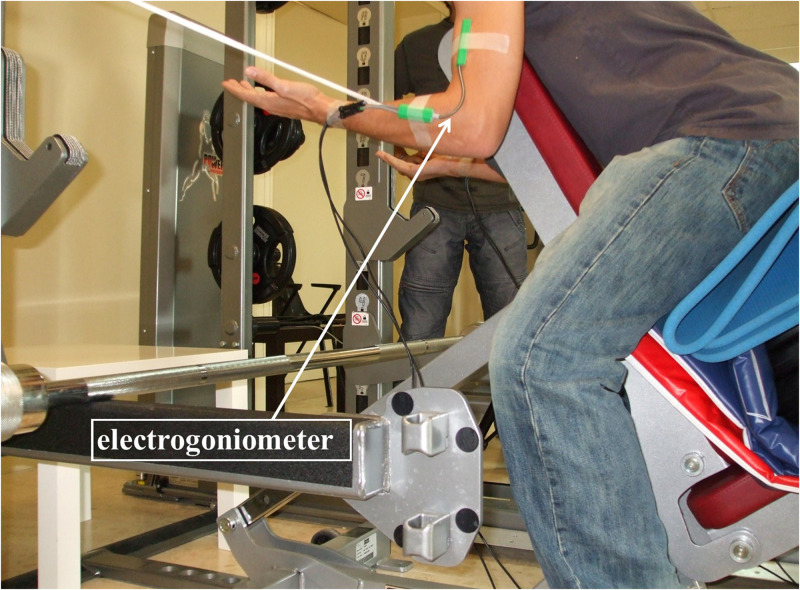
Participant in preparation for the task. The sensors of the electrogoniometer were placed on the upper arm and forearm of both extremities.

Under the framework of a network physiology approach (Bartsch et al., 2015; Ivanov et al., 2016), an interaction-dominant dynamic of the exercise-induced fatigue phenomenon is assumed (Delignières and Marmelat, 2012). Accordingly, the possibility that many component processes can lose or gain in significance during the developing fatigue, and that non-linear self-organized changes may occur in the network, is considered. In order to test the general predictions of theories of macroscopic self-organized change (e.g., Haken, 1978), some authors have already experimentally discovered the existence of critical behavior before the fatigue-induced spontaneous task disengagement. In these early studies, the elbow joint angle was treated as an order parameter, i.e., a collective control variable that macroscopically governed the activity of components of the neuromuscular axis of performers (see Figure 1). By analyzing the fatigue-induced changes in the Fourier spectra of upper limb fluctuations, Hristovski and Balagué (2010) discovered the critical phenomenon of enhanced fluctuations in the vicinity of the spontaneous task disengagement point. Subsequently, by the analysis of changes in the temporal structure of upper-limb elbow angle fluctuations, Vázquez et al. (2016) found enhanced persistent correlations in the vicinity of task disengagement, which is a hallmark of the critical slowing down phenomenon^2^ (see e.g., Koide and Maruyama, 2004; Scheffer et al., 2009, 2012; Rigamonti and Carretta, 2015). Hence, it became theoretically plausible to treat the spontaneous task disengagement as belonging to the class of non-equilibrium phase transitions. Based on the discovery of these key properties, the phenomenon of task disengagement was interpreted as a primitive, evolutionary stabilized, protective decision mechanism by which the organism spontaneously removes the cause of the perceived discomfort and the possible injury (Vázquez et al., 2016; Slapsinskaite, 2017; Pol et al., 2018).

Studying the time-variability properties of the elbow angle during a quasi-isometric exercise (Figure 1) performed until exhaustion, Vázquez et al. (2016) found a continuous evolution from an anti-persistent to a persistent time structure dynamic of the goal coordinative (i.e., order parameter) variable as fatigue developed. The authors interpreted these results as a loss of the initial fine-grained temporal control as spontaneous task failure approached. At the level of the central nervous system (CNS) spatio-temporally nested inhibition-excitation networks, which initially compete at short time scales, shift the competition toward longer-term intervals with fatigue accumulation. That is, a coalition of inhibitory effects acting at multiple nested network levels accumulate and cannot be adequately quickly compensated by excitatory intention-motivation control loops which function on longer time scales (Kiebel et al., 2008). The result is a progressively delayed possibility of adjusting the goal variable (i.e., increase of the relaxation time) that finally results in spontaneous task disengagement for a minimal additional increase of effort accumulation.

During the previously mentioned quasi-isometric exercise, both arms cooperated with compensatory movements and adjustments of the limbs to maintain the task and stabilize the control of the elbow angle (Hristovski and Balagué, 2010; Vázquez et al., 2016). Such adaptive actions included the recruitment of additional motor units and the engagement of energy transfer from other body structures to the limbs. In particular, under competitive conditions or when a real task failure is approached, a larger number of structures (from muscles to limbs) are progressively engaged with time on task or effort accumulation. The increment in the number of structures cooperating to satisfy the task goal also found that testing other coordinative variables during dynamic exercises (Balagué et al., 2014) signifies a more coherent competitive behavior in the physiological network as the task disengagement approaches. Such processes can only be identified when the motor task is prolonged enough while striving to maintain the same performance level.

The effects of effort accumulation on temporal properties of different potential coordinative collective variables (e.g., elbow joint angle, revolutions per minute during cycling, and acceleration during running) for different types of exercise have already been studied (Balagué et al., 2014; Barbosa et al., 2018; Montull et al., 2020). However, little is known about the effects of fatigue on interlimb coordination, particularly in the view of spontaneous task-disengagement. Several works have analyzed the interactions between limbs during maximum force production (Archontides and Fazey, 1993) while pedaling (Sato et al., 2019), or inter-muscular and inter-joint coordination during hammering (Côté et al., 2008), but these timeless approaches were not able to capture and explain the dynamics of the exercise-induced changes of interlimb coordination, especially to give an account on the phenomenon of fatigue-induced task disengagement.

The use of a few macroscopic variables that govern the behavior of the innumerable neuro-musculo-skeletal degrees of freedom has been shown to be a viable interpretation of the strategy that the brain-body system uses to control actions (Kelso, 1995). Thus, the search for such variables (also known as order parameters, collective, essential, or coordinative variables) is one of the key interests in motor control and learning research. While the interlimb coordination in the class of *oscillatory movements* has a long research tradition (e.g., Kelso, 1984, 1995), the interlimb coordination in *quasi-isometric* actions is still uncharted territory. For the oscillatory class of actions, the relative phase has been defined as the collective coordinative control variable. In this paper, to our knowledge for the first time, we make an attempt to define the collective variable that the brain-body system uses to coordinate the limbs in tasks that require prolonged quasi-isometric effort in order to manipulate environmental objects, such as an Olympic bar. In heterogeneous complex systems with networked interactions, such as the brain-body system, correlation (or more generally, similarity) measures have been used as order parameters (Krauth and Mézard, 1989; Parisi, 2006; Arenas et al., 2008; Hristovski et al., 2011). Hence, here we hypothesize that the brain-body system may use the same type of macroscopic action control variable in order to efficiently manipulate events and objects in the environment. More concretely, since the general prediction for complex systems is that long-range spatial correlations develop and enhance as the system approaches the tipping point (Sethna, 2006), we hypothesize that interlimb correlations will also enhance as the fatigue driven system approaches the critical point of spontaneous task disengagement.

This aim poses some methodological issues that have to be resolved first. The temporal changes in the variability of complex psychobiological time series are characterized by non-stationarity, which is not captured by traditional available techniques of analysis (e.g., frequency analysis), and thus, more sophisticated methods of non-stationarity reduction are required (Amoud et al., 2008). In addition, univariate approaches may have a limited perspective on complex fluctuations whose source is often unknown. The analysis of the simultaneously recorded data can be used to reveal the properties of underlying mechanisms: delays, loops, directed dependences. The multivariate studies can put the light on the identification of the structure of interactions in a system of multiple components (Müller et al., 2016). Such methods are useful for the causality assessment (in the Granger sense) and for understanding the information flow (Gencaga et al., 2015) between variables (by Shannon formalism). Running correlation function (RCF) and bivariate phase-rectified signal averaging (Bauer et al., 2010) methods can be used to study the interrelations between two time series recorded simultaneously. The following analysis is dedicated to the assessment of the magnitudes (strength) of temporal interrelations to reflect coordination during the task.

The aim of this study was to investigate the effect of exercise-induced fatigue on the upper interlimb coordination during a quasi-isometric exercise performed until spontaneous task disengagement. Particularly, we were interested in the possibility of defining the potential task specific order parameter that is used by performers in the goal-directed control of the brain-body-environment coordination.

## Materials and Methods

### Participants

Seven voluntary physical education students (four females and three males *M* = 22.41 years old, *SD* = 1.2) participated in the study. All of them were familiar with strength training and conditioning. Prior to taking part in the study, they completed a questionnaire to confirm their health status (Sánchez-López and Dresch, 2008). All the experimental procedures were explained to the participants before they gave their written consent for the experiment. The Local Research Ethics Committee approved the study (072015CEICEGC) according to the Helsinki Declaration.

### Procedure

**FIGURE 2:**
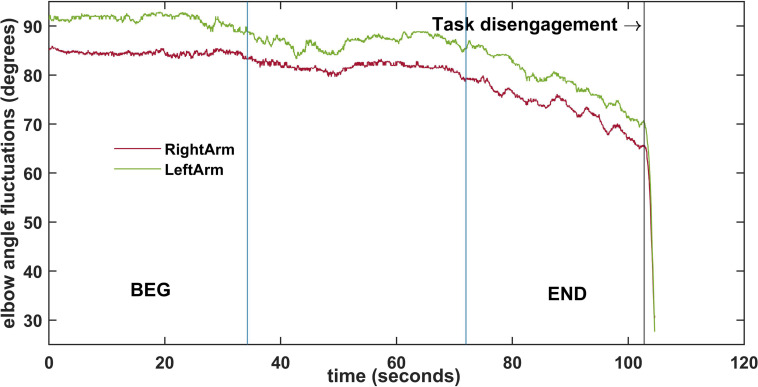
Example of the fluctuations of the elbow angle of both arms during one trial. Vertical blue lines divide the trial into three segments preceding the task disengagement. The first segment (BEG) and the last one (END) were selected for the analysis.

On three different days over a period of three weeks (one day per week), participants performed a quasi–isometric task consisting of holding an Olympic bar with 80%^3^ weight of one-repetition maximum (1RM) in an arm curl position until fatigue-induced spontaneous task failure (Figure 1). The one-repetition maximum test was performed one week prior to the start of the study to determine the maximum weight that they were able to move on a complete arm-curl exercise (M = 33.43 kg, SD = 3.16 kg). Then, 80% of the 1RM weight was calculated for each participant and used during the task. Participants were encouraged to intentionally maintain an elbow joint angle as close as they can to the initial angle of 90°. A virtual competition was organized in order to increase the likelihood that the real fatigue-induced spontaneous task disengagement was reached in all trials. Participants sat on an inclined-forward bench in order to prevent possible spinal injuries and a reference cord was placed at the level of the participant’s wrist to facilitate haptic and visual feedback on the initial position and its loss. Before the task started, the bench position and the reference cord were adjusted for each participant on every trial. The elbows of the participants were not fixed, allowing them to move freely in all three dimensions. To record the elbow angle variations, an electrogoniometer (SG110, Biometrics Ldt, Gwent, United Kingdom) was used. As shown in Figure 1, the sensors of the electrogoniometer were placed on marked points of the upper arm and forearm of both arms and were adjusted to the required starting flexion of 90°. The elbow angle variations were recorded using Ebiom software (Biometrics Ldt, Gwent, United Kingdom) for further analysis. The sampling frequency was set at 50 Hz and the amplitude resolution was 0.1 deg. for each extremity. Figure 2 shows an example of the variations of the elbow angle degrees of one participant recorded during one of the trials.

### Data Preprocessing

As the participants were unable to maintain the 90° elbow flexion throughout the trial, a trend reduction of the elbow angle series was performed before the correlation analysis. The point of spontaneous task disengagement was determined in the data sets as an abrupt and persistent switch toward negative values of the differenced time series calculated as *y* = *x* − *x*(lag = 1), where *x* denotes the elbow angle, *x*(lag = 1) the lagged elbow angle for 1 data point, and *y* denotes the angle change (Hristovski and Balagué, 2010). This negative trend was determined from a linear fit performed in overlapping windows with a 50-points length starting from the end of the recording. The fatigue-induced spontaneous task disengagement point was detected from the time series of both elbows separately. In case of any divergence between its position in the left (*L*) and right (*R*) datasets, the task disengagement point was set for the earlier position in time. The time series of both elbows after the determination of task disengagement had the same length.

The linear trend was removed from the time series by the first order polynomial fit. Subsequently, the detrended recordings were raised by the minimum of original data to obtain the referential values of the elbow angle.

As the time length to task disengagement occurrence was different between participants, the recordings were divided into three segments to compare them. The first (BEG) and the third (END) segments had the same number of points, which reflected the percentage of time evolution preceding the task disengagement. For comparison purposes the first 30% (BEG) and the last 30% (END) of the preprocessed data were selected for further analysis (see Figure 2).

### Running Correlation Function

A RCF was applied to BEG and END segments. For all calculations, a common procedure of overlapping windows was performed. The correlation coefficient was determined with respect to a predefined window width *W*. Then, the window was moved one point forward through the time series and the correlation coefficient was determined again. The procedure was repeated until the window reached the end of the selected data segment (BEG and END, separately). The described sliding approach for the temporal correlation coefficient determination is known as RCF. To obtain the limited ranges of RCF values varying between −1 and +1, the normalization by local standard deviations in the *n*-th segment σ*_*R*_(n)* and σ*_*L*_(n)* was introduced:

(1) $$R\,C\,F\,\left(n\right)=\frac{\sum_{i=1}^{W}\left(L_{n+i}-\mu_{L}\,\left(n\right)\right)\cdot \left(R_{n+i}-\mu_{R}\,\left(n\right)\right)}{\left(W-1\right)\cdot \sigma_{L}\,\left(n\right)\cdot \sigma_{R}\,\left(n\right)}.$$

In Eq. (1), μ*_*L*_(n)* and μ*_*R*_(n)* refer to the local mean determined for the left and right elbow, respectively. Note, however, that the means and standard deviations in Eq. (1) were calculated only for the current window, where left limit is *n-th* data point in the time series segment. Five windows were predefined and selected for the analysis *W* = {50, 100, 150, 200, 250, 300}, which are multiplicities of 1second due to the experimental sampling rate. Finally, we decided to verify the delays in the correlations. It was obtained by introduction of time lag *τ* in Eq. (1). The range of *τ* depends on window width *W*. The maximal *τ* was set for each case at *W/2*. The *τ* can be easily recalculated to seconds because 50 points are equal to one second. In such a realization the current windows in both signals overlapped.

For the statistical comparison between BEG and END segments, the analysis of RCF distributions was proposed. The RCF was given in the constant range < −1;1 >, and the number of data points in BEG and END segments were the same. The analysis of the correlations relies on its magnitudes in selected segments. We proposed a marker which reflected high correlations between the left and right arm, respectively. Therefore, we calculated the percentage of the RCF values which followed the rule RCF(*n*) > 0.8 in BEG and END segments separately. It was denoted as %RCF > 0.8. The constant number of data points in BEG and END segments showed that the results for each participant were not sensitive to the length of the signal.

For statistical assessment of the RCF, we determined the coefficient of variation (CV). It is defined by the quotient of standard deviation and mean values. CV is introduced in the analysis to reflect the variability and homogeneity of the marker (i.e., the percentage of the RCF > 0.8 in the two segments: BEG and END). A low value of CV indicates a small statistical dispersion of the %RCF > 0.8 marker in the studied segment.

### Bivariate Phase Rectified Signal Averaging (BPRSA) Method

In order to support the symmetric properties of the coordination represented by the RCF data analysis, the bivariate phase rectified signal averaging (BPRSA) method (originally used for the assessment of the baroreflex sensitivity; Bauer et al., 2010) was adopted. It was applied here for the estimation of the fluctuations of interrelations between the recorded data of both arms simultaneously. One recording was treated as trigger and the second as target. The changes in the target signal were estimated in accordance to increments detected in the trigger. The BPRSA computation process was modified and divided in a few successive steps:

a) The anchor points were denoted in the trigger signal and reflected in the target signal at synchronous positions in time. Anchor points were defined as values in the trigger signal which were larger than the previous ones. They were found in the sliding windows *W*, which widths were used in the RCF analysis,
b) The close surrounding of anchor points was required for further analysis: two points backward and one forward. Such surroundings for following anchor points may overlap,
c) Surroundings were aligned at anchors of target signals,
d) Averaging within the aligned surroundings was performed,
e) Finally, the marker for BPRSA was determined from four points of the averaged surroundings:

(2) $${\mathrm{BPRSA}}_{\mathrm{trigger}\to \mathrm{target}}=\frac{X\,\left(0\right)+X\,\left(1\right)-X\,\left(-1\right)-X\,\left(-2\right)}{4}$$

Points of target signal *X*(−1), *X*(−2), and *X*(1) surrounded the increase in the trigger signal in time. The increase reflected in the target signal was positioned in an anchor *X*(0). We performed computations in both possible directions, from right (trigger) to left (target) arm and in the opposite way, because the BPRSA analysis was used here to support the symmetric properties of the coordination represented by the RCF study. Therefore, we proposed a unique marker, which estimates the magnitude of fluctuations taking into account possible interrelations between data. First, the differences in the BPRSA analysis between the arms, i.e., the difference between the BPRSA (right over left) minus the BPRSA (left over right): diff(BPRSA) = BPRSA_*R*_ →_*L*_ − BPRSA_*L*_ →_*R*_, were calculated. In the next step, we estimated the ranges between 5th and 95th centiles of diff(BPRSA) values for BEG segment. We determined the diff(BPRSA) for ENG values that exceeded the centile limits given in BEG and presented them in percentages. This variable was denoted as %Range (END − BEG). Assuming larger variations in the END segment than in BEG, the expected percentages of %Range (END – BEG) should exceed 10%. Note that in such calculus, the sign of the diff(BPRSA) parameter was not taken into account. It can be treated as a single and symmetric measure of the magnitude of simultaneous fluctuations from both signals.

## Results

**FIGURE 3:**
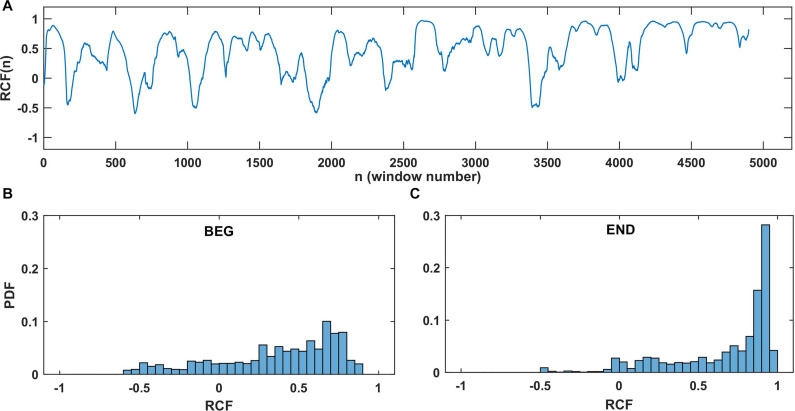
Example of RCF results for the elbow angle series from one participant in one trial. **(A)** The RCF plot of dataset until fatigue-induced spontaneous task disengagement with no time lag and window *W* = 150. **(B)** Probability distributions for RCF in the BEG segment and **(C)** probability distributions at the END segment. For better visualization, the distributions were constructed with constant number of bins equal to 40 in **(B,C)**.

**FIGURE 4:**
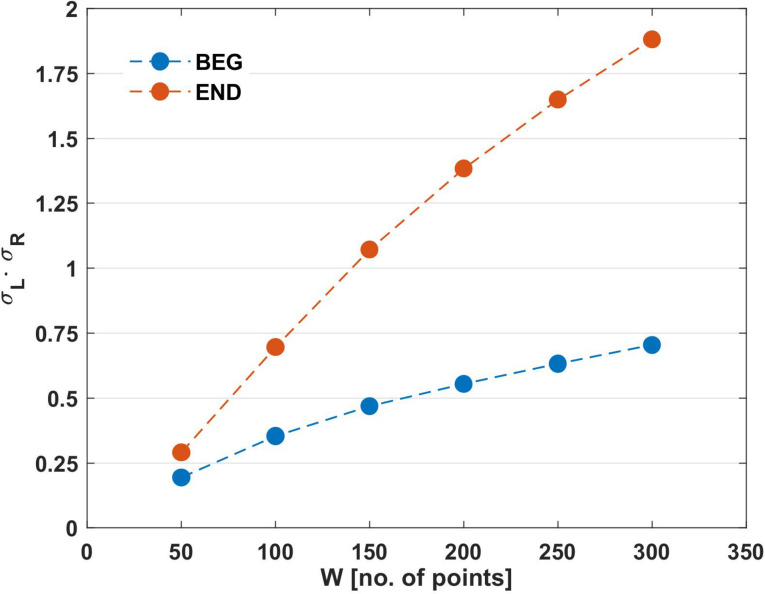
Results of mean product σ_*L*_(*W*)⋅σ_*R*_(*W*) obtained for the different analyzed windows (*W*) in the initial and final segments of the trials. BEG: initial segment of the trial and END: final segment of the trial.

**FIGURE 5:**
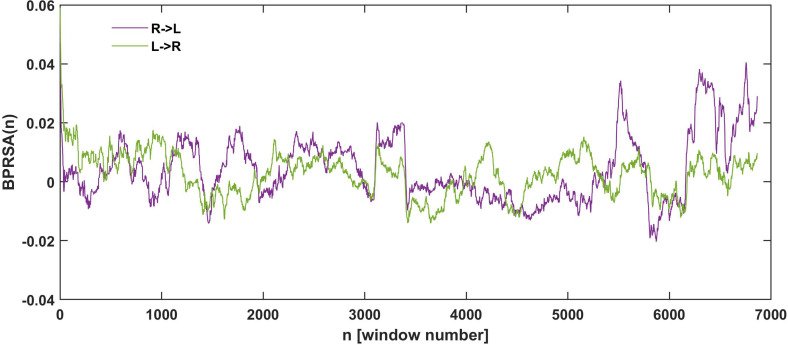
Example of adopted BPRSA results until the fatigue-induced spontaneous task disengagement for one participant with no time lag and window *W* = 300. The violet time series represents the influence of the right arm in the left arm fluctuations. Green time series represents the influence of the left arm in the right arm fluctuations. An increment in the variations between arms in the last part of the time series, close to the task disengagement, is observed.

**FIGURE 6:**
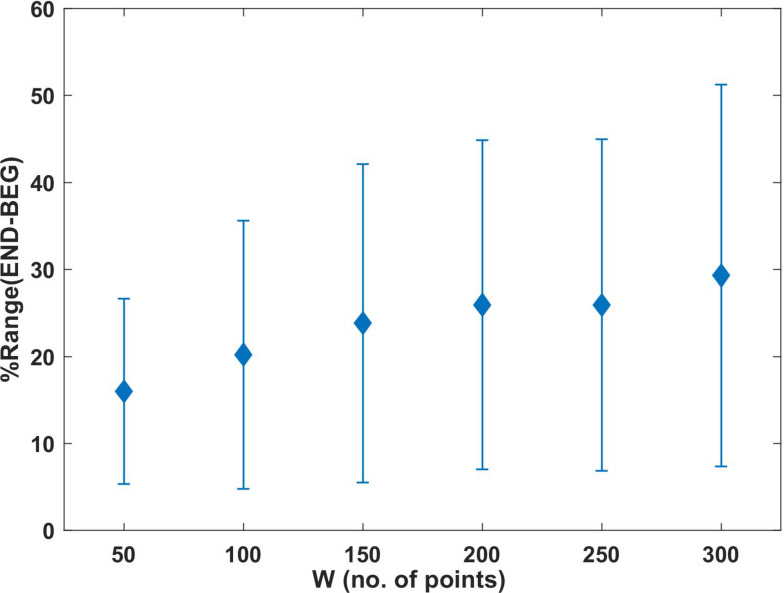
Mean of %Range (END – BEG) determined for the analyzed windows (*W*). Each point represents the value of the percentages characterizing the deviations in the END segment in relation to BEG. Error bars represent the standard deviation.

**FIGURE 7:**
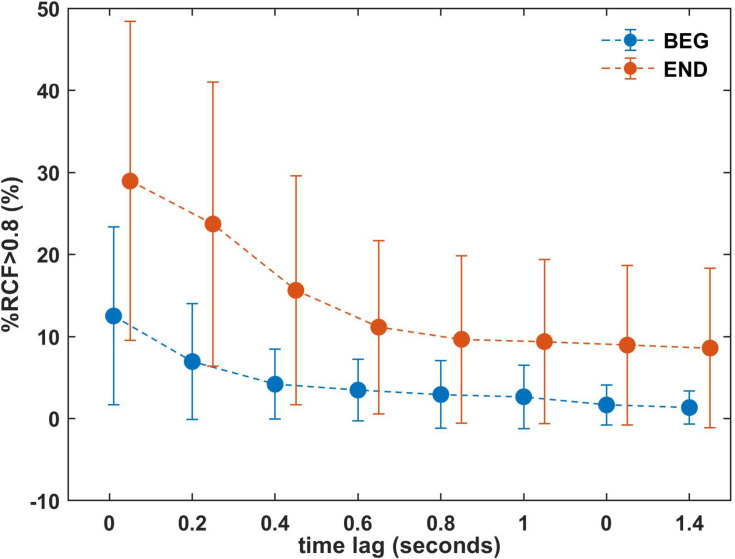
Mean %RCF > 0.8 for the BEG and END segments with increasing time lags 0, 0.2, 0.4…1.4 s for one selected window (*W* = 150). The results for the final part are shifted and dotted line is plotted to visualize the trend. Error bars represent the standard deviation. BEG: initial segment of the trial and END: final segment of the trial.

The results are divided into two sections. In the first one, the analysis of the window length in the RCF and adopted BPRSA calculus (Table 1 and Figures 3–6) are presented. In the second part, the influence of the time lag is discussed (Figure 7). In both cases, the calculations were done to BEG and END segments separately.

**TABLE 1:** The mean, SD, and CV of %RCF > 0.8 for different windows W.

Windows	50	100	150	200	250	300
Mean BEG	7.49	10.53	12.50	13.59	15.84	17.03
SD BEG	6.24	9.12	10.84	11.82	12.88	14.28
CV BEG	83.4	86.6	86.7	87.0	81.3	83.8
Mean END	17.00	26.98	28.94	29.46	28.20	27.05
SD END	8.07	14.94	19.44	21.96	23.63	25.24
CV END	47.5	55.4	67.2	74.5	83.8	93.3

Figure 3 shows an example of the RCF and its distributions in selected window *W* = 150 for one participant. It reflects the typical properties of the RCF: wide range of correlation values (positive and negative as well) and visible domination of higher RCF in the END segment than BEG segment. Each point in the plot of RCF (Figure 3A) corresponds to the correlation coefficient determined for the left and right arms datasets in the sliding window. Figures 3B,C are the histograms of RCF from the BEG and END segments. In general, we observed a domination of positive correlations. According to the marker introduced in section “Running Correlation Function,” the percentage of the high correlation magnitude (i.e., %RCF > 0.8) equals 4.6% for BEG segment and 55.1% for END segment. This means that the correlations of high magnitude (i.e., values higher than 0.8) were less probable in the BEG segment and more probable in the END segment. After determining the marker %RCF > 0.8 for each trial and each participant, the group statistics of the RCF in BEG and END segments was performed.

Table 1 shows the mean and SD of the %RCF > 0.8 marker for the windows 50, 100, 150, 200, 250, and 300. The mean of the BEG segment increased with the increment of the window length, while the mean of the END segment reached the maximum for window *W* = 200 and started to decrease for the next windows (*W* = 250 and 300). In both segments, the SD values increased with window length, but their magnitude was larger for the END segment in comparison to the BEG segment.

The results of the coefficient of variation (CV) show that the BEG segment was not sensitive to the window’s length (very small fluctuations in the third row of Table 1). However, the CV for END increased with the window’s enlargement. Note that in the latter case, the CV for *W* = 300 was twice as large than for *W* = 50. That is, while the CV was stable in the BEG segment, it increased in the END segment with the windows’ length. This can be mainly explained by the magnitude of the SD for the END segment.

To support the results of RCF and its variations in BEG and END segments (Table 1), the product σ_*L*_(*n*)⋅σ_*R*_(*n*) (Eq. 1) was calculated using the same window size. As observed in Figure 4, the mean product increased more slowly with the window’s width in the BEG segment (*W*50 = 0.19; *W*100 = 0.35; *W*150 = 0.46; *W*200 = 0.55; *W*250 = 0.63; *W*300 = 0.70) than in the END segment (*W*50 = 0.29; *W*100 = 0.69; *W*150 = 1.07; *W*200 = 1.38; *W*250 = 1.64; *W*300 = 1.80).

Figure 5 shows an example of the results for the adopted BPRSA analysis in a selected window (*W* = 300). As it is observed, the influence of the right arm on the variations of the left (violet line) and the left arm on the right (green line) increased in the last part of the task preceding the fatigue-induced task disengagement.

Figure 6 shows the mean values and SD of the proposed marker of change percentage [%Range (END − BEG)] (see section “Bivariate Phase Rectified Signal Averaging (BPRSA) Method”), which was calculated for each window (*W*50 = 16.01; *W*100 = 20.22; *W*150 = 23.83; *W*200 = 25.94; *W*250 = 25.92; *W*300 = 29.31). The results showed that the differences between END and BEG segments exceeded 10% in all windows. When %Range (END − BEG) exceeds 10%, the END segment is characterized by larger magnitude of adopted BPRSA differences than BEG. It can be interpreted as occurrence of increasing variations between arms in the last segment of effort in comparison to initial (corresponding to BEG) segments of the experiment. The SDs in Figure 6 confirms large variability between participants in adopted BPRSA determination. These results are in line with the increment obtained in the product σ_*L*_(*W*)⋅σ_*R*_(*W*) (Figure 4).

Observing the means of %RCF > 0.8 in the END segment (Table 1), we focused on the selected window *W* = 150 and introduced the time lags in temporal correlation determination (Figure 7). The lags were established at 0, 0.2, 0.4, 0.6, 0.8, 1, 1.2, and 1.4 s. The results showed a decreasing profile of %RCF > 0.8 with time lag for both segments (see Table 2). However, the larger effect of decrease was for the END segment with a relative change of 20.4%, (i.e., the difference between the time lag 0 s and the time lag 1.4 s) compared to the BEG segment, with a relative change of 11.2%. Note that the mean of %RCF > 0.8 was smaller than 10% for time lags > 0.8 s. The decrease of SD in BEG and END segments was also observed.

**TABLE 2:** Mean time lag differences in the %RCF > 0.8 marker for a window 150.

Windows (in seconds)	0	0.2	0.4	0.6	0.8	1	1.2	1.4
Mean BEG	12.50	6.94	4.18	3.47	2.93	2.65	1.66	1.34
SD BEG	10.84	7.05	4.26	3.75	4.12	3.86	2.43	2.01
Mean END	28.94	23.71	15.62	11.14	9.65	9.38	8.95	8.59
SD END	19.44	17.30	13.94	10.56	10.19	10.00	9.73	9.73

## Discussion

The interlimb coordination during a quasi-isometric exercise performed with upper extremities until spontaneous task disengagement was studied. Two different analyses were performed (RCF and adopted BPRSA) to compare the initial 30% (BEG) and final 30% (END) segments of the obtained electrogoniometry time series of both arms in different time windows. The results showed a common increment in the correlation magnitudes between both arms and between the BEG and the END segments. The findings from RCF also indicated that the level of correlations was higher on longer timescales. Based on the task goal stabilizing synergy approach (Latash, 2008; Kelso, 2009), the increase in the interlimb correlation during the final segments revealed lower interlimb independence close to task disengagement, and thus, a decreased interlimb coordination. This has been related to the impaired ability of the psychobiological system to maintain its independent control during a motor task performed until exhaustion (Vázquez et al., 2016). The increase of the correlation in the END segment of the time series, compared to the BEG segment, means that, on average, any variation in one arm is associated with variation in the same direction in the other arm. This is well observed by the values of %RCF > 0.8 (Figure 3), which indicate that the lower correlation of high magnitude and thus smaller influence between the arms were found in the BEG segment. On the contrary, higher values were found in the END segment, showing greater influence (i.e., crosstalk) between the arms, and hence, more correlation of a high magnitude.

These results show a similar development of fatigue in both limbs and are in line with previously reported results supporting the hypothesis that the task goal stabilizing synergy spreads over long time periods (Hristovski and Balagué, 2010; Vázquez et al., 2016). In both studies, the authors observed that the effort accumulation had an important role in the regulation and control of the task goal.

Previous works define fatigue as an inhibitory (neural and metabolic) protective mechanism in competition with activation, with excitatory processes acting at a neuromuscular level (Hristovski and Balagué, 2010; Balagué et al., 2014; Vázquez et al., 2016). During fatigue, this competition is produced between the intention to sustain the Olympic bar (activation process) and the loss of neuro-muscular tension (protective inhibitory process). This is manifested by the increments in the elbow angle fluctuations during the END segment: σ_*L*_(*W*)⋅σ_*R*_(*W*) (Figure 4), and %Range (END − BEG) (Figure 6). Whereas in the BEG segment these processes compete over short time scales, resulting in a stabilizing effect (i.e., small fluctuations around the task goal); as the exercise proceeds the competition gradually shifts toward longer time scales (i.e., the participants need larger periods to recover the initial elbow angle). This general mechanism may be explained by the presence of negative feedback loops, where small positive (upward) deviations from the local average, as a consequence of central excitation, is being compensated for by subsequent negative (downward) fluctuations as a result of the coupling between the inhibitory processes and the pull of gravity (Balagué et al., 2014; Vázquez et al., 2016; Montull et al., 2020). As fatigue develops, the neural, metabolic, and muscular network changes are reflected by lower muscle contractile ability due to a larger neural and metabolic inhibitory effect. Thus, as the accumulated effort increases the inhibitory influences become more prominent. To compensate for this inhibition, the larger involvement of networked supra-spinal activation processes became necessary. For example, the supra-spinal activation recruits new motor units in order to synergistically compensate for those which are already exhausted (Taylor and Gandevia, 2008). This compensatory activation is generated by processes that need larger time scales for their development and manifestation, such as the motivational processes (Kiebel et al., 2008). Hence, the competition between spatio-temporally nested inhibitory and excitatory networks of processes continuously shifts on ever-increasing time intervals (Hristovski and Balagué, 2010; Balagué et al., 2014; Vázquez et al., 2016). The time resolution of control becomes impaired, and the mutual influence (measured by RCF) between both arms rises to higher magnitudes in the END segment. Note that this process characterized by the temporal RCF marker has a dynamic profile in time (Figure 3), which is expressed even more by magnitudes of SD products (i.e., σ_*L*_(*W*)⋅σ_*R*_(*W*)) and %Range(END − BEG). As a result of this process, the mutual dependence of both limbs increases, impairing the ability of the interlimb system to flexibly negotiate the task constraints.

This increment in correlations close to the critical point is characteristic for a vast number of condensed matter and complex systems (e.g., Patashinskii and Pokrovskii, 1979). The interlimb correlations are a sort of spatial correlations (i.e., different limbs are associated with different spatial neuro-musculo-skeletal areas), which also increase as the system approaches criticality. Close to the critical point, long-range spatial correlations enhance (Sethna, 2006). Hence, our results, although not conclusive, may point to the task-specific macroscopic variable that the brain-body system uses to control the class of quasi-isometric actions. Moreover, taking into account the above, we can tentatively propose the hypothesis that at the level of brain-body multilayered networks at the critical point a process of inhibitory *percolation* takes place. In exercising biological systems, percolation has already been proposed as a self-organizing mechanism that leads to the emergence of macroscopic musculo-skeletal injuries (Pol et al., 2018). The phenomenology of task disengagement in these kinds of tasks strongly suggests the existence of a phase transition of first order (abrupt shift of the order parameter value) (Hristovski and Balagué, 2010; Balagué et al., 2014). However, in both works, it has been noted that there is also a tendency toward a drift of the stable state (elbow-angle) to lower values. This is suggestive of a system approaching the phase transition of second order (continuous shift to the new stable state). On the other hand, it is already an established fact that in multilayered complex networks both types of phase transitions may be present (Kivelä et al., 2014). These networks of the brain-body system may show various levels of fragility or robustness to targeted or random inhibitory influences depending, among others, on the structural properties of the network and its layers and to the type of potential percolation process (site, bond, or site-bond percolation) (Callaway et al., 2000). From this point of view, it would be of high importance to investigate the different models of targeted inhibition (e.g., chained inhibition of neural hubs) in the brain-body system, or randomly dispersed inhibition, and how these differ in their degree of involvement for different task constraints for this general task. It will not be a surprise if both processes compete over different time scales, and the final approach to the task disengagement depends on the type of interaction between some key personal, task, and environmental constraints (Hristovski et al., 2014).

%Range (END − BEG) within the segments represents the fluctuations of both limb’s angles and their common influence. Values exceeding 10% of %Range (END − BEG) in the END segment suggest the development of fatigue preceding task disengagement. Higher magnitudes of %Range (END − BEG) in END reflect larger sensitivity of the target to increments defined as anchor points in trigger signal. It may also reflect the loss of independence between limbs. Furthermore, the results of the CV (Table 1) showed a constant mean increase with window enlargement, suggesting the preservation of correlation between left and right arms in the BEG segment when the impact of fatigue development was low. On the contrary, the correlation magnitude was larger for END in all studied windows. Our findings corroborate previous results showing that larger fluctuations and variability (i.e., deviations from the average elbow angle) increase in all participants close to the fatigue-induced spontaneous task disengagement (Hristovski and Balagué, 2010). According to the authors, the enhancement in the elbow angle variability is related to an increased activity of the neuromuscular system constrained to find new functional synergies as a result of the initial elbow-angle destabilization provoked by the effort accumulation.

The time lags introduced in the analysis corroborate the last observations and indicate that the high levels of correlation between left and right arms measured by %RCF > 0.8 are short term, and is confirmed by the sudden drop observed for time lag < 0.8 s. This higher magnitude in time lag < 0.8 s reflected the quick adjustments and reconfigurations made by participants to maintain the task goal. However, we observed that for the longer time lags (τ = 1, 1.2, 1.4 s) the %RCF > 0.8 decreased very slowly and was characterized by smaller SD. These findings support the notion that the correlation between limbs is observed and produced over shorter time scales, due to the small adjustments that participants made to maintain the elbow angle. However, this effect was not observed for larger lags.

In summary, the dynamics within the upper limb system became increasingly critical, pointing toward a mutually aligned, more coherent behavior between the cooperative and competitive processes within the neuromuscular axis (Balagué et al., 2014). The smaller magnitudes of RCF and adopted BPRSA for the BEG segment meant potential for more independent and flexible spatio-temporal control of the coordinative variable, i.e., elbow angle (Vázquez et al., 2016). On the contrary, the higher values of the %RCF > 0.8 and a higher percentage in the %Range (END − BEG) for the END segment indicated a more rigid control of the task. We demonstrate that close to exhaustion, the interlimb system becomes excessively coupled compared to the beginning of the exercise where the independent, more refined, control of the upper limbs is possible. Furthermore, the enhancement in the correlation and mutual dependence between the arms can be proposed as a new marker of the approaching exhaustion.

The results of this study can help to understand the dynamics of the correlation between different systems under the presence of constraints and strengthen previous research findings about the different strategies that participants use to negotiate fatigue. Our findings demonstrate that the accumulation of effort cannot be explained by simple, component-dominant approaches but by an integrative, network interaction-dominant approach of the phenomenon. However, caution should be taken in the interpretation of the results because the values of the SD found in the larger windows warn that the last observation is only valid for lower windows width (<200). The higher amplitude of the elbow angle fluctuations during the last part of the exercise observed in the time series, and the different strategies made by the participants to maintain the exercise until spontaneous task disengagement, could explain this observation. Further investigations are needed to study how the correlation between different psychobiological signals is modified due to effort accumulation.

From a practical point of view, as the enhancement of fluctuations is connected phenomenologically to interlimb correlations, some effort phases can be detected during quasi-isometric types of exercises. These phases, which may be used with training and rehabilitation purposes, may help to detect the phases of stable and metastable states of effort, and predict with a high likelihood the spontaneous task disengagement. The first phase is characterized by lower interlimb correlations and lower fluctuations; the second is characterized by enhanced interlimb correlations and fluctuations. The third is characterized by an excessive increase of these two markers of fatigue and the vicinity of the spontaneous task disengagement (Balagué et al., 2014). Thus, practitioners can control the exercise volume through such markers to attain their training or rehabilitation purposes. Exercise volumes that include only the first phase may be used when no significant neuro-muscular restructuring is planned, for example, in warm-up or recovery sessions. The volumes, including the middle phase, may be used for fostering larger intra and interlimb coordinative restructuring, and exercise volumes (including the last phase) may be included when more drastic reconfigurations and adaptations are planned.

In conclusion, the study reveals an increment of the interlimb correlation of upper extremities during a quasi-isometric exercise performed until fatigue-induced spontaneous task disengagement. The increment of interlimb correlations close to the critical task disengagement point suggests analogies with critical processes in other networked systems (e.g., Arenas et al., 2008). This points to the possibility that the RCF may be the macroscopic variable that the brain-body system uses for coordination and control of the vast number of neuro-musculo-skeletal degrees of freedom during quasi-isometric classes of action. The bivariate analyses showed that the developed fatigue influenced the coordination between the arms, resulting in a loss of their initial autonomy to control the task. The enhancement of the interlimb correlation at the end of the task showed a more aligned, mutually influenced behavior between the limbs as a consequence of fatigue. The results also point toward the use of bivariate methods of analysis to assess the correlation between different psychobiological signals that fluctuate during exercise.

## Data Availability Statement

The raw data supporting the conclusions of this article will be made available by the authors, without undue reservation.

## Ethics Statement

The studies involving human participants were reviewed and approved by Comité d’Ètica d’Investigacions Cliniques de l’Administració de Catalunya. The patients/participants provided their written informed consent to participate in this study.

## Author Contributions

PV, RH, and NB conceived and designed the experiments. PV and NB performed the experiments. MP data analysis and presentation of the results. PV, RH, NB, and MP interpreted the results, contributed to reagents, materials, analysis tools, and wrote the manuscript. All authors contributed to the article and approved the submitted version.

## Conflict of Interest

The authors declare that the research was conducted in the absence of any commercial or financial relationships that could be construed as a potential conflict of interest.

## Footnotes

1 In line with a network physiology of exercise approach, in this paper the common term “muscle fatigue” was replaced by the term “exercise-induced fatigue” to avoid centering the muscle as the cause of fatigue.

2 Critical slowing down is a phenomenon that arises as a consequence of the increased relaxation time of the system toward its stable state after a perturbation has been applied. The outcome of the slower relaxation toward the stable state is the enhancement of persistent temporal correlations between subsequent fluctuations (see e.g., Koide and Maruyama, 2004; Scheffer et al., 2009, 2012; Rigamonti and Carretta, 2015).

3 The task constraint of 80% of 1-RM was applied because it provided a sufficient data sample and guaranteed the emergence of task disengagement in a short testing time.

## References

[B1] Amoud H. Snoussi H. Hewson D. Duchêne J. (2008). Univariate and bivariate empirical mode decomposition for postural stability analysis. *EURASIP J. Adv. Signal Proc.* 2008:657391 10.1155/2008/657391

[B2] Archontides C. Fazey J. A. (1993). Interlimb interactions and constraints in the expression of máximum forcé: a review, some implications and suggested underlying mechanisms. *J. Sports Sci.* 11 145–158. 10.1080/02640419308729978 8497017

[B3] Arenas A. Díaz-Guilera A. Kurths J. Moreno Y. Zhou C. (2008). Synchronization in complex networks. *Phys. Rep.* 469 93–153.

[B4] Balagué N. Hristovski R. Vainoras A. Vázquez P. Aragonés D. (2014). “Psychobiological integration during exercise,” in *Complex Systems in Sport*, eds Davids K. Hristovski R. Araújo D. Balagué N. Button C. Passos P. (New York, NY: Routedgle), 62–81.

[B5] Barbosa T. M. Chen S. Morais J. E. Costa M. J. Batalha N. (2018). The changes in classical and nonlinear parameters after maximal bout to elicit fatigue in competitive swimming. *Hum. Mov. Sci.* 58 321–329. 10.1016/j.humov.2017.12.010 29249572

[B6] Bartsch R. P. Liu K. K. L. Bashan A. Ivanov P. C. (2015). Network physiology: how organ systems dynamically interact. *PLoS One* 10:e0142143. 10.1371/journal.pone.0142143 26555073 PMC4640580

[B7] Bauer A. Morley-Davies A. Barthel P. Müller A. Ulm K. Malik M. (2010). Bivariate phase-rectified signal averaging for assessment of spontaneous baroflex sensitivity: pilot study of the technology. *J. Electrocardiol.* 43 649–653. 10.1016/j.jelectrocard.2010.05.012 20638668

[B8] Callaway D. S. Newman M. E. Strogatz S. H. Watts D. J. (2000). Network robustness and fragility: percolation on random graphs. *Phys. Rev. Lett.* 85 5468–5471. 10.1103/physrevlett.85.5468 11136023

[B9] Côté J. N. Feldman A. G. Mathieu P. A. Levin M. (2008). Effects of fatigue on intermuscular coordination during repetitive hammering. *Motor Control* 12 79–92. 10.1123/mcj.12.2.79 18483444

[B10] Delignières D. Marmelat V. (2012). Fractal fluctuations and complexity: current debates and future challenges. *Crit. Rev. Biomed. Eng*. 40 485–500. 10.1615/CritRevBiomedEng.2013006727 23356693

[B11] Enoka R. M. Duchateau J. (2016). Translating fatigue to human performance. *Med. Sci. Sports Exerc*. 48 2228–2238. 10.1249/MSS.0000000000000929 27015386 PMC5035715

[B12] Gandevia S. C. (2001). Spinal and supraspinal factors in human muscle fatigue. *Physiol. Rev.* 81 1725–1789. 10.1152/physrev.2001.81.4.1725 11581501

[B13] Gencaga D. Knuth K. H. Rossow W. B. (2015). A recipe for the estimation of information flow in a dynamical system. *Entropy* 17 438–470. 10.3390/e17010438

[B14] Haken H. (1978). Synergetics. *Phys. Bull.* 28:412.

[B15] Hristovski R. Balagué N. (2010). Fatigue-induced spontaneous termination point–Nonequilibrium phase transitions and critical behavior in quasi-isometric exertion. *Hum. Mov. Sci.* 29 483–493. 10.1016/j.humov.2010.05.004 20619908

[B16] Hristovski R. Balagué N. Schöllhorn W. (2014). “Basic notions in the science of complex systems and nonlinear dynamics,” in *Complex systems in Sport*, eds Davids K. Hristovski R. Araújo D. Balagué N. Button C. Passos P. (London: Routledge), 3–17.

[B17] Hristovski R. Davids K. Araújo D. Passos P. (2011). Constraints-induced emergence of functional novelty in complex neurobiological systems: a basis of creativity in sport. *Nonlinear Dynamics Psychol. Life Sci.* 15 175–206. 21382260

[B18] Ivanov P. C. Liu K. K. L. Bartsch R. P. (2016). Focus on the emerging new fields of network physiology and network medicine. *New J. Phys.* 18:100201 10.1088/1367-2630/18/10/100201 PMC6415921 30881198

[B19] Kelso J. A. S. (1984). Phase transitions and critical behavior in human bimanual coordination. *Am. J. Physiol.* 246 1000–1004. 10.1152/ajpregu.1984.246.6.R1000 6742155

[B20] Kelso J. A. S. (1995). *Dynamic Patterns: The Self-Organization of Brain and Behavior.* Cambridge, MA: MIT press.

[B21] Kelso J. A. S. (2009). “Synergies: atoms of brain and behavior,” in *Progress in Motor Control*, ed. Sternad D. (NewYork, NY: Springer Science+Business Media), 83–91. 10.1007/978-0-387-77064-2_5

[B22] Kiebel S. J. Daunizeau J. Friston K. J. (2008). A hierarchy of time-scales and the brain. *PLoS Comput. Biol.* 4:e1000209. 10.1371/journal.pcbi.1000209 19008936 PMC2568860

[B23] Kivelä M. Arenas A. Barthelemy M. Gleeson J. P. Moreno Y. Porter M. A. (2014). Multilayer networks. *J. Complex Netw.* 2 203–271.

[B24] Koide T. Maruyama M. (2004). Enhancement of critical slowing down in chiral phase transition—Langevin dynamics approach. *Nucl. Phys. A* 742 95–129. 10.1016/j.nuclphysa.2004.06.013

[B25] Krauth W. Mézard M. (1989). Storage capacity of memory networks with binary couplings. *J. Phys.* 50 3057–3066. 10.1051/jphys:0198900500200305700

[B26] Latash M. (2008). *Synergy.* Oxford: Oxford University Press.

[B27] Montull L. Vázquez P. Rocas L. Hristovski R. Balagué N. (2020). Flow as an embodied state. Informed awareness of slackline walking. *Front. Psychol.* 10:2993. 10.3389/fpsyg.2019.02993 31998205 PMC6968164

[B28] Müller A. Kraemer J. F. Penzel T. Bonnemier H. Kurths J. Wessel N. (2016). Causality in physiological signals. *Physiol. Meas.* 37 R46–R72. 27100820 10.1088/0967-3334/37/5/R46

[B29] Parisi G. (2006). Spin glasses and fragile glasses: statics, dynamics, and complexity. *Proc. Natl. Acad. Sci. U.S.A.* 103 7948–7955. 10.1073/pnas.0601120103 16690744 PMC1459408

[B30] Patashinskii A. Z. Pokrovskii V. L. (1979). *Fluctuation Theory of Phase Transitions.* New York, NY: Pergamon Press.

[B31] Pol R. Hristovski R. Medina D. Balagué N. (2018). From microscopic to macroscopic sports injuries. Applying the complex dynamic systems approach to sports medicine: a narrative review. *Br. J. Sports Med*. 53 1214–1220. 10.1136/bjsports-2016-097395 29674346

[B32] Rigamonti A. Carretta P. (eds). (2015). “Phase diagrams, response functions and fluctuations,” in *Structure of Matter. UNITEXT for Physics*, (Cham: Springer).

[B33] Sánchez-López M. D. P. Dresch V. (2008). The 12-item general health questionnaire (GHQ-12): reliability, external validity and factor structure in the Spanish population. *Psicothema* 4 839–843. 18940092

[B34] Sato T. Shigetome S. Tokuyasu T. (2019). “Inter-limb muscle coordination induced by fatigue during pedaling,” in *Proceedings of the 37th International Society of Biomechanics in Sport Conference*, Oxford, OH, 153–156.

[B35] Scheffer M. Bascompte J. Brock W. A. Brovkin V. Carpenter S. R. Dakos V. (2009). Early-warning signals for critical transitions. *Nature* 461 53–59. 10.1038/nature08227 19727193

[B36] Scheffer M. Carpenter S. R. Lenton T. M. Bascompte J. Brock W. A. Dakos V. (2012). Anticipating critical transitions. *Science* 338 344–348. 10.1126/science.1225244 23087241

[B37] Sethna J. (2006). *Statistical Mechanics: Entropy, Order Parameters, and Complexity*, Vol. 14 Oxford: Oxford University Press.

[B38] Slapsinskaite A. (2017). *Exercise-Induced Pain. Dynamic Perspective.* Doctoral dissertation, University of Barcelona, Barcelona.

[B39] Taylor J. L. Gandevia S. C. (2008). A comparison of central aspects of fatigue in submaximal and maximal voluntary contractions. *J. Appl. Physiol.* 104 542–550. 10.1152/japplphysiol.01053.2007 18032577

[B40] Vázquez P. Hristovski R. Balagué N. (2016). The path to exhaustion: time-variability properties of coordinative variables during continuous exercise. *Front. Physiol.* 7:37. 10.3389/fphys.2016.00037 26913006 PMC4753307

